# Assessment and validation of the pet-owner relationship scale for Brazil

**DOI:** 10.3389/fpsyg.2024.1412451

**Published:** 2024-06-12

**Authors:** Luis Felipe Dias Lopes, Eduarda Grando Lopes, Mauren Pimentel Lima, Fillipe Grando Lopes, Daniela Pegoraro, Rosangela de Arruda Saragozo, Thais Ribeiro Lauz, Joana Vieira dos Santos

**Affiliations:** ^1^Center for Social and Human Sciences, Postgraduate Program in Administration, Federal University of Santa Maria, Santa Maria, Brazil; ^2^Rural Science Center, Degree in Veterinary Medicine, Federal University of Santa Maria, Santa Maria, Brazil; ^3^Center for Health Sciences, Postgraduate Program in Nursing, Federal University of Santa Maria, Santa Maria, Brazil; ^4^Ernesto Dornelles Hospital, Porto Alegre, Brazil; ^5^Center for Social and Human Sciences, Postgraduate Program in Psychology, Federal University of Santa Maria, Santa Maria, Brazil; ^6^Psychology Research Center, Faculty of Human and Social Sciences, University of Algarve, Faro, Portugal

**Keywords:** dog, cat, pet, cross-cultural adaptation, pet-owner interactions

## Abstract

**Introduction:**

This study aimed to perform a cross-cultural adaptation of the cat-owner/dog-owner relationship scales. The method involved several stages: conceptual, item, semantic, operational, measurement, and functional equivalence. Procedures included translation, synthesis of translations, back-translation, consensus on the English versions, external evaluation by the original authors, expert committee evaluation, and pre-tests.

**Methods:**

The study surveyed 234 pet owners across Brazil using a 20-item questionnaire. Data analysis utilized confirmatory factor analysis, covariance-based modeling, and multigroup analysis.

**Results:**

The study confirmed the content and construct validity of the model, demonstrating good convergent validity. Hypotheses testing revealed significant inverse relationships between Perceived Cost and Perceived Emotional Closeness, and between Perceived Cost and Pet-Owner Interactions. A positive correlation was found between Perceived Emotional Closeness and Pet-Owner Interactions, with Perceived Emotional Closeness also mediating the relationship between Perceived Cost and Pet-Owner Interactions. No significant differences were found across different pet owner groups, indicating the scale’s invariance and reliability across various demographics.

**Discussion:**

The study significantly expands understanding of the complex dynamics in pet-owner relationships and emphasizes the interplay between emotional and practical factors. It offers valuable insights for future research and practices in animal and human welfare.

## 1 Introduction

The relationship between people and their pets has been a significant societal aspect for centuries. Initially centered on nurturing, this relationship has evolved into one characterized by companionship and affection (Menache, 1998; Serpell, 2000). Brazil is renowned for its rich cultural heritage, encompassing diverse traditions and customs that vary expressively from region to region. Despite this variability of cultures, there is a unifying thread that runs through Brazilian society—the profound bond between pet owners and their animals. Whether it’s with dogs, cats, or other pets, this relationship is universally valued and cherished across the nation.

According to Instituto Pet Brasil (2022), over 150 million Brazilians relate with their pets in loving and affectionate ways. This interaction transcends traditional limits, becoming integral to the social and emotional fabric of the Brazilian populace. Recognizing pets’ roles in their owners’ lives is vital for a deeper understanding of the intimacy of these relationships (Riggio et al., 2021).

The presence of animals in human lives not only enhances health and promotes psychological well-being but also contributes to increased longevity. This phenomenon, known as the “pet effect,” underscores the significant roles pets play in reducing stress, lowering blood pressure, and providing essential emotional support (Allen, 2003).

One commonly utilized theoretical framework to understand the positive impacts of human-animal companionship is Attachment Theory, which suggests that humans inherently possess a need for attachment or a sense of belonging to someone (Bowlby, 1977). This perspective indicates that pets can fulfill this need, serving as attachment figures and sources of emotional security. Research has indicated that individuals with a profound attachment to their pets may perceive minimal distinctions between interactions with animals and humans, highlighting the depth of these human-animal bonds (Kurdek, 2008).

The link between pet ownership and the provision of social support holds particular significance for older individuals, including those who are single, divorced, remarried, or without children present, as they often exhibit higher levels of attachment to pets and are more likely to anthropomorphize them (Albert and Bulcroft, 1988). Additionally, Taniguchi et al. (2018) found that caring for a dog or cat can be an effective health promotion strategy to increase physical activity and facilitate social participation among older adults. Pet ownership has been shown to be related to lower levels of depressive symptoms (Taniguchi et al., 2018) and anxiety (Bolstad et al., 2021).

Despite the benefits, the evidence regarding the association between pet ownership and subjective well-being remains unclear; some studies found no differences in the proportion of pet owners and non-owners who described themselves as “very happy” (e.g., Herzog, 2011).

A recent systematic review (Kretzler et al., 2022) indicated that social isolation may be associated with pet ownership, while loneliness is less likely. However, the study also concluded that there is a scarcity of research examining the association between pet ownership, loneliness, and social isolation in low- and middle-income countries.

Given this context, this study sought to adapt the cat-owner/dog-owner relationship scales for measuring affectivity in pet-owner relationships. Howell et al. (2017) initially proposed these scales for cats, with adaptations for dogs by Riggio et al. (2021). The Pet-Owner Relationship Scale (PORS) will be modified for both dog and cat owners in Brazil, which is in line with the global effort to recognize and quantify the significance of pets, particularly dogs and cats, for individuals’ mental and emotional health.

Pets provide companionship and emotional support, invaluable for people whether individuals are living alone or coping with occupational illnesses (Foltin and Glenk, 2023), owning pets, such as dogs and cats, indirectly promotes physical activity through activities such as daily walks, grooming, and veterinarian visits. These interactions contribute significantly to the physical and mental well-being of their owners. Research has consistently shown that petting a dog or a cat can lower stress levels and blood pressure, promoting relaxation and overall well-being (Teo et al., 2022). Moreover, dogs and cats play a crucial role in enhancing public health and population well-being by fostering social interactions and strengthening bonds between individuals, as well as between animals and people (Wells et al., 2022).

This article details the process of cross-culturally adapting the scales proposed by Howell et al. (2017) for cats and Riggio et al. (2021) for dogs to the Brazilian context. The questionnaire has been translated into Swedish (Handlin et al., 2012); Spanish (Calvo et al., 2016); German (Schöberl et al., 2015); Danish (Meyer and Forkman, 2014); and Dutch (van Houtert et al., 2019). In addition to Howell scale, it is known that other researchers used similar scales, for example, Lexington Attachment to Pets (LAPS), original scale (Johnson et al., 1992); Mexican (Ramírez et al., 2014); Italian (Uccheddu et al., 2019); Germany (Hielscher et al., 2019); and Brazil (Albuquerque et al., 2023).

**Figure 1:**
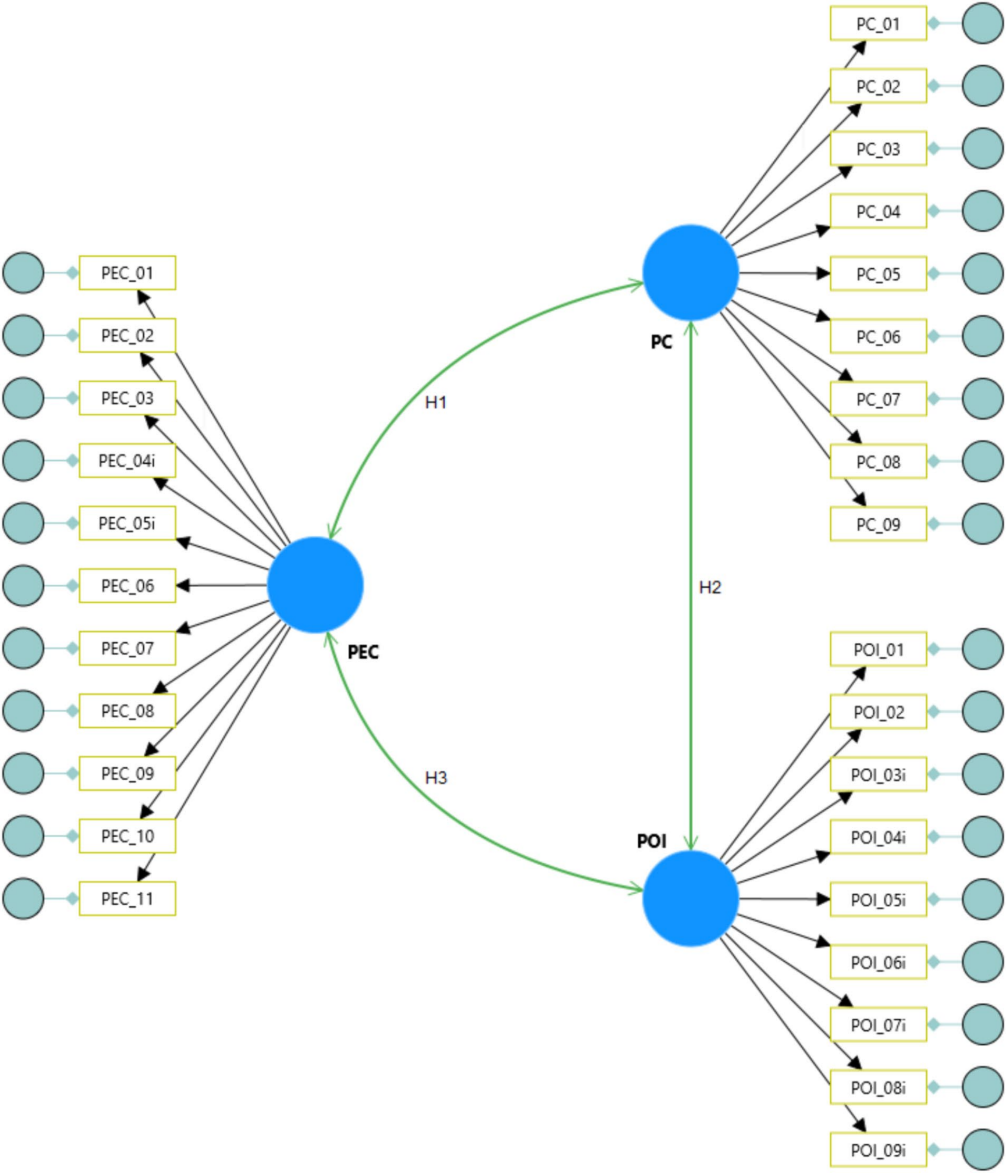
The initial measurement model proposed by Howell et al. (2017). PC, Perceived cost, POI, pet-owner interactions, PEC, perceived emotional closeness.

By employing a comprehensive and culturally sensitive method, this study aims to provide a reliable scale for researchers, animal health professionals, and pet owners, enhancing the understanding of the pet-owner relationship’s dynamics and depth in Brazil. This study’s significance lies in the growing number of pet owners globally and the diverse roles pets play in Brazilian households. Pets are companions for the lonely, integral family members for households with children and the elderly, and sources of emotional support, promoting mental and physical health and enriching their owners’ daily lives (Ward et al., 2023). Moreover, dogs and cats play a crucial role in enhancing public health and population well-being by fostering social interactions and strengthening bonds between individuals, as well as between animals and people. These questionnaires will be completed by pet owners, providing valuable insights into the dynamics of the human-animal relationship. Hence, this research seeks to pave the way for future studies and interventions that benefit both owners and pets, underscoring the importance of this relationship in public health and social well-being. The initial measurement model, based on the scale proposed by Howell et al. (2017), is presented in Figure 1.

To elucidate the potential positive or negative relationships within the model’s dimensions, the following hypotheses were established to provide context.

We initially posited that the relationship between Perceived Cost and Emotional Closeness is inversely proportional. Perceived Cost, encompassing financial, time, physical, and emotional investments, negatively impacts an owner’s emotional closeness toward their pet. This could stem from the burdens of high costs, potentially leading to feelings of overload or stress, thereby affecting the owner’s emotional connection with the pet (Barcelos et al., 2023).

*H1*: Perceived cost is negatively related to perceived emotional closeness.

There is a suggested negative correlation between Perceived Cost and Pet-Owner Interactions. Higher Perceived Costs associated with pet care are believed to result in less frequent or lower-quality interactions between the owner and the pet. This may be because owners who perceive higher costs may feel less inclined or able to engage frequently or positively with their pets (Brown, 2018; Merkouri et al., 2022; Kipperman, 2023).

*H2*: Perceived cost is negatively related to pet-owner interactions.

Conversely, a positive relationship is anticipated between Perceived Emotional Closeness and Pet-Owner Interactions. It is assumed that the stronger the emotional bond an owner feels toward their pet, the more frequent and meaningful their interactions will be. A robust emotional connection typically fosters a greater desire to spend time with the pet, enhancing the quality and frequency of interactions for both the owner and the pet (Borrelli et al., 2022; Junça-Silva et al., 2022; Somppi et al., 2022).

*H3*: Perceived emotional closeness is related to pet-owner interactions.

The study further posits that Perceived Emotional Closeness may act as a mediator between Perceived Cost and Pet-Owner Interactions. Even in the presence of high Perceived Costs, a strong emotional bond can mitigate these costs, leading to sustained or increased interaction with the pet. This suggests that pet owners who share a deeper emotional connection with their pets may be more resilient to the challenges associated with pet care (Collins et al., 2022; Barcelos et al., 2023; Hoy et al., 2023).

*H4*: Perceived emotional closeness mediates the relationship between perceived cost and pet-owner interactions.

## 2 Materials and methods

This research employed a descriptive, comparative cross-sectional design with a quantitative approach. The study gathered data from a diverse cohort of pet owners spanning various professions and geographic regions in Brazil, including students, educators, healthcare professionals, law enforcement officers, civil servants, and workers from other sectors. Each participant had a distinct relationship with their pet.

Data were collected using online questionnaires using Google Forms and disseminated between September and November 2023 via social networks such as Facebook, Instagram, LinkedIn, and WhatsApp. Participation was contingent upon informed consent obtained after a thorough briefing on the study’s objectives. This research was conducted in strict adherence to ethical guidelines governing human subject research and secured approval from the Ethics Committee (CAAE no. 44261821.8.0000.5346, opinion no. 4.606.946).

### 2.1 Participants

The study recruited 234 pet owners through convenience sampling. Eligibility criteria included being over 18 years old and owning a pet. As listed in Table 1, the demographic breakdown of survey participants was as follows: 76.1% (*n* = 178) were female, 34.2% (*n* = 80) aged 18–30 years, 53.8% (*n* = 126) were married or in a long-term relationship, 38.5% (*n* = 90) had at least two household members, 48.3% (*n* = 113) held or were pursuing graduate degrees, and 82.1% (*n* = 192) resided in the southern region of Brazil. Most respondents (51.7%, *n* = 121) lived exclusively with dogs, 27.8% (*n* = 65) with both dogs and cats, and 20.5% (*n* = 48) solely with cats. Among dog-only households, 28.2% had only one dog, while 14.1% of cat-only households had a single cat. In households with both dogs and cats, a higher prevalence (12.8%) of having four or more pets was noted.

**Table 1:** Social and demographic characteristics of the participants (*n* = 234).

Demographic data	*n*	%
Sex		
Female	178	76.1
Male	56	23.9
Age (years)		
18–31	80	34.2
31–40	45	19.2
41–50	50	21.4
>50	59	25.2
Marital status		
Married	126	53.8
Single	91	38.9
Divorced/Widowed	17	7.3
Level of education	46	19.7
High school education	75	32
Higher education	113	48.3
Graduate education		
Region of Brazil		
South	200	85.5
Southeast	11	4.7
Central West	20	8.5
North and Northeast	3	1.3
Household composition (no. of people)		
1	30	12.8
2	90	38.5
3	58	24.8
≥4	56	23.9
Household pet		
Dog(s)	121	51.7
Cat(s)	48	20.5
Dog(s) and cat(s)	65	27.8
Number of household pets		
1	90	38.5
2	59	25.2
3	35	15
≥4	50	21.3

### 2.2 Measure

The scale adaptation for this study involved a panel of five esteemed animal health experts. These professionals evaluated and subsequently tailored the indicators to align with the Portuguese language and the context of dog and cat ownership. The original scale, conceptualized by Howell et al. (2017), comprises 3 (three) key dimensions and 29 (twenty-nine) indicators:

- Perceived cost (PC), encompassing 9 (nine) indicators, gages the owner’s perceived financial burden associated with pet ownership.
- Perceived emotional closeness (PEC), with 11 (eleven) indicators, delves into the depth of the emotional bond between the pet owner and their animal, a critical factor in the overall quality of the relationship.
- Pet-Owner Interactions (POI), featuring 9 (nine) indicators, quantitatively assesses the day-to-day interactions between the pet and its owner, including activities like play, grooming, and providing companionship. This dimension offers invaluable insights into the practical nuances of pet-owner relationships. The scale, refined through careful statistical analysis, is presented in the Appendix.

### 2.3 Data analysis

This study utilized the Statistical Package for the Social Sciences (version 26.0) to evaluate the reliability and validity of the measurement model derived from the original framework. The conceptual model underwent a thorough examination, leveraging the principal fit indicators common in confirmatory factor analysis (CFA), as noted by Shrestha (2021). Additionally, the model’s applicability was assessed using SmartPLS software (version 4.1.0.0), employing covariance-based structural equation modeling as outlined by Ringle et al. (2022).

The Kruskal-Wallis non-parametric test was employed to discern and compare the behavioral patterns across different pet owner groups. This test was instrumental in identifying any notable disparities among the groups. Furthermore, a multigroup analysis was conducted to determine the model’s invariance and consistency across varied owner demographics.

## 3 Results and discussion

Step-by-step structural equation modeling (CB-SEM) to cross-culturally validate a scale: (1) metric invariance and reliability and validity assessment (Tables 2, 3); (2) residual invariance (Figure 1 and Table 4); and (3) report the results of hypothesis testing (Table 5); (4) compare latent means to explore cultural differences (Table 6); and (5) discuss the implications of cultural differences or similarities found in the study (Collier, 2020).

**Table 2:** Dimensions, indicators, commonalities, cronbach’s alpha, composite reliability, and average variance extracted.

Dimensions/indicators*	*h* ^2^	KMO	θ (CA)	θ (CR)	AVE
Perceived Cost (PC)		0.90	0.67	0.67	0.53
PC02	0.64				
PC03	0.66				
PC04	0.70				
PC05	0.76				
PC06	0.61				
Perceived Emotional Closeness (PEC)		0.76	0.82	0.82	0.58
PEC01	0.69				
PEC02	0.66				
PEC03	0.72				
PEC06	0.83				
PEC07	0.86				
PEC08	0.82				
PEC09	0.76				
PEC10	0.89				
PEC11	0.62				
Pet-Owner Interactions (POI)		0.80	0.88	0.89	0.59
POI01	0.77				
POI02	0.72				
POI03i	0.81				
POI04i	0.69				
POI05i	0.70				
POI06i	0.84				

*h*^2^, commonality; KMO, Kaiser-Meyer-Olkin measure; CA, Cronbach’s alpha; CR, composite reliability; AVE, average variance extracted; i, inverse evaluation.

* The data of the indicators are provided in Appendix A.

**Table 3:** Fornell-Larcker and Heterotrait-Monotrait ratio.

Dimensions	$\sqrt{AVE}$	Pearson’s correlation matrix		
		PC	POI	PEC
PC	0.73	1.00		
POI	0.77	−0.34	1.00	
PEC	0.77	−0.42	0.57	1.00
		HTMT		
		---		
POI		0.44	---	
PEC		0.52	0.64	---

AVE, Average variance extracted; PC, perceived cost; POI, pet-owner interactions; PEC, perceived emotional closeness.

**Table 4:** Test of adequacy of the proposed model.

Models	χ^2^	df	χ^2^/df	RMSEA	SRMR	GFI	CFI	NFI	AGFI
Acceptable fit	---	---	< 3	< 0.10	< 0.05	> 0.90	> 0.90	> 0.90	> 0.90
Structural model	414.71	167.00	2.48	0.09	0.04	0.96	0.92	0.96	0.94

*χ*^2^, Chi-square, df: degrees of freedom; *χ*^2^/df, Chi-square to degrees of freedom ratio; RMSEA, root mean square error of approximation; SRMR, standardized root mean square residual; GFI, goodness of fit index; CFI, comparative fit index; NFI, normed fit index; AGFI, adjusted goodness of fit index.

**Table 5:** Analysis of structural coefficients.

Hypotheses	Direct relationships	*β*	sd	*t*-statistic (*β*/sd)	*p*
H1	PC → PEC	−0.11	0.05	2.30	0.006
H2	PC → POI	−0.11	0.04	2.01	0.011
H3	PEC → POI	0.44	0.02	3.56	0.000
	Indirect relationship (mediation)				
H4	PC → PEC → POI	0.10	0.03	2.944	0.001

PC, Perceived cost; POI, pet-owner interactions; PEC, perceived emotional closeness; *β*, beta coefficient; sd, standard deviation.

**Table 6:** Comparative analysis of dimensions between types of owners.

Dimensions	Dogs (*n* = 121)	Cats (*n* = 48)	Dogs and cats (*n* = 65)	p*
PC	2.5 (0.76)	2.5 (0.75)	2.6 (0.72)	0.585
PEC	4.1 (0.82)	4.1 (0.72)	3.9 (0.88)	0.080
POI	4.6 (0.74)	4.8 (0.66)	4.7 (0.52)	0.237

Values are reported as mean and standard deviation (in parentheses). PC, Perceived cost; POI, pet-owner interactions; PEC, perceived emotional closeness.

* Kruskal-Wallis test.

The initial stage involved conduction out an Exploratory Factor Analysis (EFA) to evaluate the commonalities of the indicators and then an CFA to validate the dimensional structures of the scale. This analysis verified which indicators effectively measured the dimensions, thus confirming the content and construct validity of the model based on participant responses. When the varimax rotation technique was applied, indicators with commonalities (*h^2^*) below 0.6 were excluded. The Kaiser-Meyer-Olkin measure for all three dimensions surpassed 0.7, suggesting suitability for further analysis (Hair et al., 2017).

Furthermore, Cronbach’s alpha (CA), composite reliability (CR), and average variance extracted (AVE) were assessed. These metrics aligned with standards set by Hair et al. (2017) (0.65 < θ < 0.95 and AVE > 0.5), indicating a consistent relationship between dimensions and indicators and demonstrating the model’s good convergent validity. As a result of the (EFC), some indicators were excluded from the initial model (Table 2).

For discriminant validity assessment, the Fornell-Larcker criterion and Heterotrait-Monotrait ratio (HTMT) were utilized (Table 3). Pearson’s correlation analysis revealed that the square root of the lowest AVE (0.729) exceeded the highest correlation between dimensions (PEC vs. POI = 0.57), positioned below the main diagonal (Fornell and Larcker, 1981). Below the main diagonal, HTMT values were below 0.9 (Henseler et al., 2015). These findings indicate that the model satisfactorily met the measurement validation criteria.

**Figure 2:**
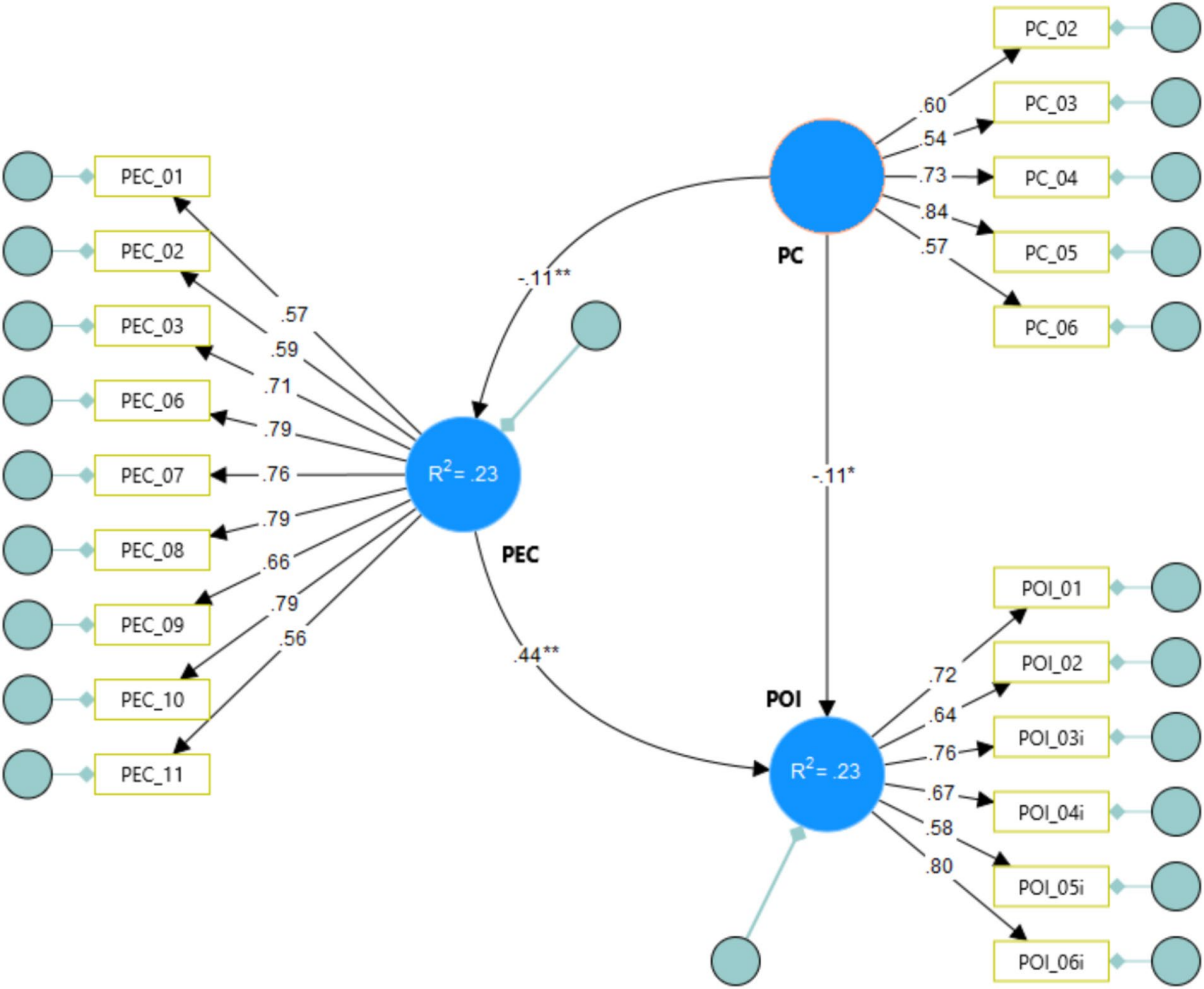
Final structural equation model. *p < 0.05; **p < 0.01. PC, Perceived Cost; PEC, Perceived Emotional Closeness; and POI, Pet-Owner Interactions.

Figure 2 illustrates the structural relationships between the model’s dimensions. Factorial loads are presented by the arrows linking the dimension (circle) with the indicators (rectangles), which statistically should contain *p* < 0.01, meaning they are significant for the model. Conversely, linking one dimension to another presents structural coefficients and their significances; hypotheses will be confirmed when *p* < 0.01 and *p* < 0.01. Within predictive dimensions, explanation coefficients are presented along with their respective significances.

Table 4 details the model’s fit quality. The results indicate a robust fit, evidenced by the chi-square test (*χ*^2^ = 414.71), degrees of freedom (df = 167), chi-square to degrees of freedom ratio (*χ*^2^/df = 2.48), root mean square error of approximation (RMSEA = 0.09), comparative fit index (CFI = 0.92), and standardized root mean square residual (SRMR = 0.05) (Henseler et al., 2015).

Table 5 validates the proposed hypotheses. It confirms a significant inverse relationship between Perceived Cost and Perceived Emotional Closeness (H1) and between Perceived Cost and Pet-Owner Interaction (H2). Hypothesis H3 delineates the positive correlation between Perceived Emotional Closeness and Pet-Owner Interaction. Hypothesis H4 posits that Perceived Emotional Closeness mediates the relationship between Perceived Cost and Pet-Owner Interaction.

Table 6 shows that for the three dimensions there were no significant differences (*p* > 0.05) between the types of owners, so it can be said that the indicators behave uniformly and homogeneously between the groups.

A CFA was conducted using the scale of Howell et al. (2017), which originally included three dimensions and 29 indicators. Post analysis, the scale was refined to 20 indicators, distributed as 5 for PC, 9 for PEC, and 6 for POI. Table 5 support the hypotheses in the general context. The validation of Hypotheses 1 and 2 indicates an inverse relationship of PC with both PEC and POI. This suggests that the burdens of cost may impede the development of a strong emotional bond and active engagement with pets, a notion corroborated by several studies (Borrelli et al., 2022; Junça-Silva et al., 2022; Merkouri et al., 2022; Somppi et al., 2022; Kipperman, 2023).

Understanding these dynamics is key to enhancing the well-being of pets and their owners, potentially informing strategies to improve their relationship. The confirmation of Hypothesis 3, which posits a positive relationship between PEC and POI, underscores the significance of emotional bonds in human-animal relationships. It suggests that stronger emotional connections lead to more frequent and higher-quality interactions, a conclusion supported by various research findings (Borrelli et al., 2022; Junça-Silva et al., 2022; Somppi et al., 2022). From both ethological and psychological viewpoints, these findings highlight affection as a crucial factor in fostering positive human-animal interactions (Boissy et al., 2007; Rault et al., 2020; Pongrácz and Dobos, 2023).

Hypothesis 4 reveals that PEC acts as a mediator in the relationship between PC and pet interactions, implying that a strong emotional bond can alleviate the negative effects of high PC on interaction levels. These insights suggest that reinforcing emotional connections between owners and pets could be viable for maintaining or enhancing interactions, regardless of the associated costs. This positive mediation signifies that PEC intensifies the influence of PC on POI (Ogata et al., 2023). Hence, a stronger emotional bond can effectively negate the deterring impact of PC on an owner’s willingness to engage with their pet (Collins et al., 2022; Barcelos et al., 2023; Hoy et al., 2023). These validated hypotheses lay a solid scientific groundwork for a deeper understanding of the complexities inherent in pet-owner relationships, emphasizing the interplay between emotional and practical aspects. This knowledge serves as a foundation for future research, public policies, and practices aimed at enhancing the well-being of pets and their owners.

## 4 Conclusion

Based on the proposed objective and the scale’s validity, this study provides a deeply informed and scientifically grounded understanding of the relationship between pet owners and their pets. Adapting the scale proposed by Howell et al. (2017) and Riggio et al. (2021) for Brazilian dog and cat owners has proven to be psychometrically sound. The confirmatory factor analysis preserved many original indicators, thereby demonstrating the scale’s robustness.

As for the scale’s invariance, the absence of significant differences in coefficients among various types of pet owners (dogs vs. cats vs. dogs and cats) indicates that the scale is consistent across these groups in the Brazilian context, where a wide and culturally significant variety of pets is present. This suggests that the scale is reliable and valid for measuring constructs related to the pet-owner relationship in Brazil, irrespective of the type of pet. It is important to highlight that specific cultural and socioeconomic factors in Brazil may influence this relationship, underscoring the need for a contextualized analysis to ensure the accuracy and applicability of the results.

The findings of this study offer valuable insights for society and public health administration in devising practical and effective strategies to promote the well-being of both pets and their owners. By understanding the intricate dynamics of pet-owner relationships, administrators can implement targeted interventions aimed at strengthening the emotional connection between them. This could involve initiatives such as: - Establishing pet-friendly policies; Providing access to affordable veterinary care; Promoting responsible pet ownership; and supporting mental health initiatives.

Administrations can allocate resources for mental health support services targeted at pet owners experiencing emotional distress or difficulties in their relationship with their pets. This includes providing access to counseling, support groups, and stress management programs tailored to the unique challenges of pet ownership.

By incorporating these evidence-based strategies into public health policies and initiatives, administrations can effectively address the complex interplay between pet ownership and human well-being, ultimately fostering healthier and more resilient communities.

According to Herzog (2011), the study of human-animal interactions is interesting, important, and challenging. It remains unclear whether and under what circumstances pets make people happier and healthier. However, it is evident that animals play a role in nearly every aspect of human psychological and cultural life. Therefore, this scale contributes to a better understanding of the relationship between humans and their pets in daily life.

### 4.1 Limitations and future research

As limitations, the results of this study may not be generalizable to all pet-owner groups due to reliance on a convenience sample. Additionally, the accuracy of psychometric scales, particularly when adapted to different cultures or populations, may vary. Nevertheless, the scale demonstrated evidence of validity within the Brazilian context, reinforcing its applicability and relevance.

As a suggestion for future research, it is suggested to assess how pets’ behavior af-fects the owner-pet relationship, as *integrating* behavioral assessments of pets could offer insights into how pets’ behavior influences the relationship dynamics, including aspects of emotional closeness and perceived costs.

## Data availability statement

The raw data supporting the conclusions of this article will be made available by the authors, without undue reservation.

## Ethics statement

The studies involving humans were approved by Ethics Committee of Federal University of Santa Maria. The studies were conducted in accordance with the local legislation and institutional requirements. The participants provided their written informed consent to participate in this study. Written informed consent was obtained from the individual(s) for the publication of any potentially identifiable images or data included in this article.

## Author contributions

LL: Writing – review & editing, Writing – original draft, Visualization, Validation, Supervision, Software, Resources, Project administration, Methodology, Investigation, Formal analysis, Data curation, Conceptualization. EL: Writing – review & editing, Investigation, Conceptualization. ML: Writing – original draft, Validation, Formal analysis, Conceptualization. FL: Formal analysis, Writing – original draft, Investigation, Conceptualization. DP: Writing – original draft, Validation, Conceptualization, Methodology, Investigation. RS: Writing – review & editing, Methodology, Investigation. TL: Methodology, Investigation, Writing – review & editing. JS: Writing – review & editing, Validation, Conceptualization.

## References

[ref1] Albert A. Bulcroft K. (1988). Pets, families and the life course. J. Marriage Fam. 50, 543–552. doi: 10.2307/352019

[ref2] Albuquerque N. S. Costa D. B. Reis Rodrigues G. Sessegolo N. S. Moret-Tatay C. Irigaray T. Q. (2023). Adaptation and psychometric properties of Lexington attachment to pets scale: Brazilian version (LAPS-B). J. Vet. Behav. 61, 50–56. doi: 10.1016/j.jveb.2022.12.005

[ref3] Allen K. (2003). Are pets a healthy pleasure? The influence of pets on blood pressure. Curr. Dir. Psychol. Sci. 12, 236–239. doi: 10.1046/j.0963-7214.2003.01269.x

[ref4] Barcelos A. M. Kargas N. Maltby J. Mills D. S. (2023). Potential psychosocial explanations for the impact of pet ownership on human well-being: evaluating and expanding current hypotheses. Hum. Anim. Interact. 2023:8. doi: 10.1079/hai.2023.0008

[ref5] Boissy A. Manteuffel G. Jensen M. B. Moe R. O. Spruijt B. Keeling L. J.. (2007). Assessment of positive emotions in animals to improve their welfare. Physiol. Behav. 92, 375–397. doi: 10.1016/j.physbeh.2007.02.003 17428510

[ref6] Bolstad C. J. Porter B. Brown C. J. Kennedy R. E. Nadorff M. R. (2021). The relation between pet ownership, anxiety, and depressive symptoms in late life: propensity score matched analyses. Anthrozoös 34, 671–684. doi: 10.1080/08927936.2021.1926707, PMID: 34776606 PMC8580122

[ref7] Borrelli C. Riggio G. Howell T. J. Piotti P. Diverio S. Albertini M.. (2022). The cat–owner relationship: validation of the Italian C/DORS for cat owners and correlation with the LAPS. Animals 13:69. doi: 10.3390/ani13010069, PMID: 36611680 PMC9817682

[ref8] Bowlby J. (1977). The making and breaking of affectional bonds: II. Some principles of psychotherapy: the fiftieth Maudsley lecture (expanded version). Br. J. Psychiatry 130, 421–431. doi: 10.1192/bjp.130.5.421, PMID: 861423

[ref9] Brown B. R. (2018). The dimensions of pet-owner loyalty and the relationship with communication, trust, commitment and perceived value. Vet. Sci. 5:95. doi: 10.3390/vetsci5040095, PMID: 30404210 PMC6313907

[ref10] Calvo P. Bowen J. Bulbena A. Tobeña A. Fatjó J. (2016). Highly educated men establish strong emotional links with their dogs: a study with Monash dog owner relationship scale (MDORS) in committed Spanish dog owners. PLoS One 11:e0168748. doi: 10.1371/journal.pone.0168748, PMID: 28033397 PMC5199054

[ref12] Collier J. (2020). Applied structural equation modeling using AMOS: basic to advanced techniques. New York: Routledge.

[ref13] Collins C. Haase D. Heiland S. Kabisch N. (2022). Urban green space interaction and wellbeing–investigating the experience of international students in Berlin during the first COVID-19 lockdown. Urban For. Urban Green. 70:127543. doi: 10.1016/j.ufug.2022.127543, PMID: 35291447 PMC8913405

[ref14] Foltin S. Glenk L. M. (2023). Current perspectives on the challenges of implementing assistance dogs in human mental health care. Vet. Sci. 10:62. doi: 10.3390/vetsci10010062 36669063 PMC9867308

[ref15] Fornell C. Larcker D. F. (1981). Evaluating structural equation models with unobservable variables and measurement error. J. Mark. Res. 18, 39–50. doi: 10.1177/002224378101800104

[ref16] Hair J. F. Hult G. T. M. Ringle C. Sarstedt M. (2017). A primer on partial least squares structural equation modeling (PLS-SEM). Los Angeles: Sage Publications.

[ref17] Handlin L. Nilsson A. Ejdebäck M. Hydbring-Sandberg E. Uvnäs-Moberg K. (2012). Associations between the psychological characteristics of the human–dog relationship and oxytocin and cortisol levels. Anthrozoös 25, 215–228. doi: 10.2752/175303712X13316289505468

[ref9001] Herzog H. (2011). The impact of pets on human health and psychological well-being: fact, fiction, or hypothesis?. Current directions in psychological science. 20, 236–239. doi: 10.1177/0963721411415220

[ref18] Henseler J. Ringle C. M. Sarstedt M. (2015). A new criterion for assessing discriminant validity in variance-based structural equation modeling. J. Acad. Mark. Sci. 43, 115–135. doi: 10.1007/s11747-014-0403-8

[ref19] Hielscher B. Gansloßer U. Froböse I. (2019). Attachment to dogs and cats in Germany: translation of the Lexington attachment to pets scale (LAPS) and description of the pet owning population in Germany. Hum. Anim. Interact. Bull. 2019, 1–18. doi: 10.1079/hai.2019.0006

[ref20] Howell T. J. Bowen J. Fatjó J. Calvo P. Holloway A. Bennett P. C. (2017). Development of the cat-owner relationship scale (CORS). Behav. Process. 141, 305–315. doi: 10.1016/j.beproc.2017.02.024, PMID: 28279780

[ref21] Hoy L. S. Stangl B. Morgan N. (2023). Dog guardians’ subjective well-being during times of stress and crisis: A diary study of affect during COVID-19. People Anim. 6:164. doi: 10.13140/RG.2.2.10095.00164

[ref22] Instituto Pet Brasil. (2022). Censo Pet IPB: com alta recorde de 6% em um ano, gatos lideram crescimento de animais de estimação no Brasil. Available at: https://institutopetbrasil.com/fique-pordentro/amor-pelos-animais-impulsiona-os-negocios-2-2/.

[ref23] Johnson T. P. Garrity T. F. Stallones L. (1992). Psychometric evaluation of the Lexington attachment to pets scale (LAPS). Anthrozoös 5, 160–175. doi: 10.2752/089279392787011395

[ref24] Junça-Silva A. Almeida M. Gomes C. (2022). The role of dogs in the relationship between telework and performance via affect: a moderated moderated mediation analysis. Animals 12:1727. doi: 10.3390/ani12131727, PMID: 35804626 PMC9264855

[ref25] Kipperman B. (2023). The influence of economics on decision-making. Practice 77:55. doi: 10.1002/9781119986355.ch8

[ref26] Kretzler B. König H. H. Hajek A. (2022). Pet ownership, loneliness, and social isolation: a systematic review. Soc. Psychiatry Psychiatr. Epidemiol. 57, 1935–1957. doi: 10.1007/s00127-022-02332-9 35816194 PMC9272860

[ref9002] Kurdek L. A. (2008). Pet dogs as attachment figures. Journal of social and personal relationships. 25, 247–266.

[ref27] Menache S. (1998). Dogs and human beings: a story of friendship. Soc. Anim. 6, 67–86. doi: 10.1163/156853098x00069

[ref28] Merkouri A. Graham T. M. O’Haire M. E. Purewal R. Westgarth C. (2022). Dogs and the good life: a cross-sectional study of the association between the dog–owner relationship and owner mental wellbeing. Front. Psychol. 13:903647. doi: 10.3389/fpsyg.2022.903647, PMID: 35923726 PMC9341998

[ref29] Meyer I. Forkman B. (2014). Dog and owner characteristics affecting the dog–owner relationship. J. Vet. Behav. 9, 143–150. doi: 10.1016/j.jveb.2014.03.002

[ref30] Ogata N. Weng H. Y. Messam L. L. (2023). Temporal patterns of owner-pet relationship, stress, and loneliness during the COVID-19 pandemic, and the effect of pet ownership on mental health: a longitudinal survey. PLoS One 18:e0284101. doi: 10.1371/journal.pone.0284101, PMID: 37099517 PMC10132673

[ref31] Pongrácz P. Dobos P. (2023). What is a companion animal? An ethological approach based on Tinbergen’s four questions. Crit. Rev. 10:1284869. doi: 10.3389/fvets.2023.1284869, PMID: 38026638 PMC10656766

[ref32] Ramírez M. T. G. Quezada Berumen L. D. C. Hernández R. L. (2014). Psychometric properties of the Lexington attachment to pets scale: Mexican version (LAPS-M). Anthrozoös 27, 351–359. doi: 10.2752/175303714X13903827487926

[ref33] Rault J. L. Waiblinger S. Boivin X. Hemsworth P. (2020). The power of a positive human–animal relationship for animal welfare. Front. Vet. Sci. 7:590867. doi: 10.3389/fvets.2020.590867, PMID: 33240961 PMC7680732

[ref34] Riggio G. Piotti P. Diverio S. Borrelli C. Di Iacovo F. Gazzano A.. (2021). The dog–owner relationship: refinement and validation of the Italian C/DORS for dog owners and correlation with the LAPS. Animals 11:2166. doi: 10.3390/ani11082166, PMID: 34438624 PMC8388506

[ref35] Ringle C. M. Wende S. Becker J. -M. (2022). SmartPLS 4. Oststeinbek: SmartPLS GmbH.

[ref36] Schöberl I. Beetz A. Solomon J. Gee N. Kotrschal K. (2015). Social factors influencing cortisol modulation in dogs during a strange situation procedure. J. Vet. Behav. 11, 77–85. doi: 10.1016/j.jveb.2015.09.007

[ref37] Serpell J. A. (2000). “Domestication and history of the cat” in The domestic cat: the biology of its behaviour. eds. Turner D. C. Bateson P. (Cambridge: Cambridge University Press), 180–192.

[ref38] Shrestha N. (2021). Factor analysis as a tool for survey analysis. Am. J. Appl. Math. Stat. 9, 4–11. doi: 10.12691/ajams-9-1-2

[ref39] Somppi S. Törnqvist H. Koskela A. Vehkaoja A. Tiira K. Väätäjä H.. (2022). Dog–owner relationship, owner interpretations and dog personality are connected with the emotional reactivity of dogs. Animals 12:1338. doi: 10.3390/ani12111338, PMID: 35681804 PMC9179432

[ref40] Taniguchi Y. Seino S. Nishi M. Tomine Y. Tanaka I. Yokoyama Y.. (2018). Physical, social, and psychological characteristics of community-dwelling elderly Japanese dog and cat owners. PLoS One 13:e0206399. doi: 10.1371/journal.pone.0206399, PMID: 30427858 PMC6241120

[ref41] Teo J. T. Johnstone S. J. Römer S. S. Thomas S. J. (2022). Psychophysiological mechanisms underlying the potential health benefits of human-dog interactions: A systematic literature review. Int. J. Psychophysiol. 180, 27–48. doi: 10.1016/j.ijpsycho.2022.07.007, PMID: 35901904

[ref42] Uccheddu S. De Cataldo L. Albertini M. Coren S. Da Graça Pereira G. Haverbeke A.. (2019). Pet humanisation and related grief: development and validation of a structured questionnaire instrument to evaluate grief in people who have lost a companion dog. Animals 9:933. doi: 10.3390/ani9110933, PMID: 31703410 PMC6912713

[ref43] van Houtert E. A. Endenburg N. Wijnker J. J. Rodenburg T. B. van Lith H. A. Vermetten E. (2019). The translation and validation of the Dutch Monash dog–owner relationship scale (MDORS). Animals 9:249. doi: 10.3390/ani9050249 31100924 PMC6562642

[ref44] Ward C. Johnson I. Bamwine P. Light M. (2023). The pet paradox: uncovering the role of animal companions during the serious health events of people experiencing homelessness. Anthrozoös 37, 343–359. doi: 10.1080/08927936.2023.2280376

[ref45] Wells D. L. Clements M. A. Elliott L. J. Meehan E. S. Montgomery C. J. Williams G. A. (2022). Quality of the human–animal bond and mental wellbeing during a COVID-19 lockdown. Anthrozoös 35, 847–866. doi: 10.1080/08927936.2022.2051935

