# Supplementary material for: Assessment and validation of the pet-owner relationship scale for Brazil
Source: Front Psychol. 2024 Jun 12;15:1412451. doi: 10.3389/fpsyg.2024.1412451 (PMC11199857; doi:10.3389/fpsyg.2024.1412451)
Supplement: Supplementary file 1 [file Table_1.pdf]

Appendix A

Table A1. The questionnaire applied to Brazilian pet-owners.
(English and Portuguese for the purpose of this article).

Escala de Relacionamento Pet-Tutor (ERPT)

PEP = **Proximidade Emocional Percebida** (Perceived Emotional Closeness - PEC)
CP = **Custo Percebido** (Perceived Cost - PC)
IPT = **Interação Pet-Tutor** (Pet-Owner Interactions - POI)

Item	Questions	Variable
1	**Meu PET me dá motivo para me levantar de manhã.**<br>(My pet gives me a reason to get up in the morning)<br>**(1) Discordo totalmente (2) Discordo (3) Nem concordo nem discordo (4) Concordo (5) Concordo totalmente**<br>(1) Strongly disagree (2) Disagree (3) Neither agree nor disagree (4) Agree (5) Strongly agree	PEC01
2	**Há aspectos importantes de ter um PET que eu não goste.**<br>There are important aspects of having a pet that I do not like.<br>**(1) Discordo totalmente (2) Discordo (3) Nem concordo nem discordo (4) Concordo (5) Concordo totalmente**<br>(1) Strongly disagree (2) Disagree (3) Neither agree nor disagree (4) Agree (5) Strongly agree	PC02
3	**Com que frequência você beija seu PET?**<br>How often do you kiss your pet?<br>**(1) Nunca (2) Uma vez por mês (3) Uma vez por semana (4) Uma vez a cada 3 dias (5) Pelo menos uma vez por dia**<br>(1) Never (2) Once a month (3) Once a week (4) Once every 3 days (5) At least once a day	PEC02
4	**Eu gostaria que meu PET e eu nunca tivéssemos que estar separados.**<br>I wish my pet and I never had to be apart.<br>**(1) Discordo totalmente (2) Discordo (3) Nem concordo nem discordo (4) Concordo (5) Concordo totalmente**<br>(1) Strongly disagree (2) Disagree (3) Neither agree nor disagree (4) Agree (5) Strongly agree	PEC03
5	**Meu PET faz muita bagunça.**<br>My pet makes a lot of mess.<br>**(1) Discordo totalmente (2) Discordo (3) Nem concordo nem discordo (4) Concordo (5) Concordo totalmente**<br>(1) Strongly disagree (2) Disagree (3) Neither agree nor disagree (4) Agree (5) Strongly agree	PC03
6	**Com que frequência você brinca com seu PET?**<br>How often do you play with your pet?<br>**(1) Nunca (2) Uma vez por mês (3) Uma vez por semana (4) Uma vez a cada 3 dias (5) Pelo menos uma vez por dia**<br>(1) Never (2) Once a month (3) Once a week (4) Once every 3 days (5) At least once a day	POI01
7	**Incomoda-me que meu PET me impeça de fazer coisas que eu gostava antes de adotá-lo.**<br>It bothers me that my pet prevents me from doing things I enjoyed before I adopted it.<br>(1) Strongly disagree (2) Disagree (3) Neither agree nor disagree (4) Agree (5) Strongly agree	PC04
8	**Com que frequência você passa o tempo observando seu PET?**<br>How often do you spend time watching your pet?	POI02

	**(1) Nunca (2) Uma vez por mês (3) Uma vez por semana (4) Uma vez a cada 3 dias (5) Pelo menos uma vez por dia**<br>(1) Never (2) Once a month (3) Once a week (4) Once every 3 days (5) At least once a day	
9	**É desagradável que às vezes eu tenha que mudar meus planos por causa do meu PET.**<br>I find it unpleasant that sometimes I have to change my plans because of my pet.<br>**(1) Discordo totalmente (2) Discordo (3) Nem concordo nem discordo (4) Concordo (5) Concordo totalmente**<br>(1) Strongly disagree (2) Disagree (3) Neither agree nor disagree (4) Agree (5) Strongly agree	PC05
10	**Meu PET gera custos altos para meu orçamento.**<br>My pet adds significant expenses to my budget.<br>**(1) Discordo totalmente (2) Discordo (3) Nem concordo nem discordo (4) Concordo (5) Concordo totalmente**<br>(1) Strongly disagree (2) Disagree (3) Neither agree nor disagree (4) Agree (5) Strongly agree	PC06
11	**Com que frequência você conversa com seu PET?**<br>How often do you talk to your pet?<br>**(1) Pelo menos uma vez por dia (2) Uma vez a cada 3 dias (3) Uma vez por semana (4) Uma vez por mês (5) Nunca**<br>(1) At least once a day (2) Once every 3 days (3) Once a week (4) Once a month (5) Never	POI03i*
12	**Gostaria de ter meu PET perto de mim o tempo todo.**<br>I want to have my pet near me all the time.<br>**(1) Discordo totalmente (2) Discordo (3) Nem concordo nem discordo (4) Concordo (5) Concordo totalmente**<br>(1) Strongly disagree (2) Disagree (3) Neither agree nor disagree (4) Agree (5) Strongly agree	PEC06
13	**Se as pessoas me deixassem, meu PET sempre estaria comigo.**<br>If people left me, my pet would always be with me.<br>**(1) Discordo totalmente (2) Discordo (3) Nem concordo nem discordo (4) Concordo (5) Concordo totalmente**<br>(1) Strongly disagree (2) Disagree (3) Neither agree nor disagree (4) Agree (5) Strongly agree	PEC07
14	**Meu PET me ajuda a passar por momentos difíceis.**<br>My pet helps me through difficult times.<br>**(1) Discordo totalmente (2) Discordo (3) Nem concordo nem discordo (4) Concordo (5) Concordo totalmente**<br>(1) Strongly disagree (2) Disagree (3) Neither agree nor disagree (4) Agree (5) Strongly agree	PEC08
15	**Com que frequência você abraça seu PET?**<br>How often do you hug your pet?<br>**(1) Pelo menos uma vez por dia (2) Uma vez a cada 3 dias (3) Uma vez por semana (4) Uma vez por mês (5) Nunca**<br>(1) At least once a day (2) Once every 3 days (3) Once a week (4) Once a month (5) Never	POI04i*
16	**Meu PET me proporciona companhia constante.**<br>My pet provides me with constant companionship.<br>**(1) Discordo totalmente (2) Discordo (3) Nem concordo nem discordo (4) Concordo (5) Concordo totalmente**<br>(1) Strongly disagree (2) Disagree (3) Neither agree nor disagree (4) Agree (5) Strongly agree	PEC09
17	**Com que frequência você tem seu PET com você enquanto relaxa?**<br>How often do you have your pet with you while you relax?	POI05i*

This is a provisional file, not the final typeset article

	**(1) Pelo menos uma vez por dia (2) Uma vez a cada 3 dias (3) Uma vez por semana (4) Uma vez por mês (5) Nunca**<br>(1) At least once a day (2) Once every 3 days (3) Once a week (4) Once a month (5) Never	
18	**Meu PET está por perto sempre que preciso ser consolado.**<br>My pet is always around whenever I need to be comforted.<br>**1) Discordo totalmente (2) Discordo (3) Nem concordo nem discordo (4) Concordo (5) Concordo totalmente**<br>(1) Strongly disagree (2) Disagree (3) Neither agree nor disagree (4) Agree (5) Strongly agree	PEC10
19	**Quão traumático você acha que será para você quando seu PET morrer?**<br>How traumatic do you think it will be for you when your pet dies?<br>(1) Muito não traumático (2) Não traumático (3) Nem traumático nem não traumático (4) Traumático (5) Muito traumático<br>(1) Very non-traumatic (2) Non-traumatic (3) Neither (4) Traumatic (5) Very traumatic	PEC11
20	**Com que frequência você acaricia seu PET?**<br>How often do you pet your pet?<br>(1) Pelo menos uma vez por dia (2) Uma vez a cada 3 dias (3) Uma vez por semana (4) Uma vez por mês (5) Nunca<br>(1) At least once a day (2) Once every 3 days (3) Once a week (4) Once a month (5) Never	POI06i*

PEC: Perceived emotional closeness, PC: perceived cost, POI: pet-owner interactions. *Questions with "i = inverse" should be reversed when analyzed.
